# A nomogram model for predicting risk factors and the outcome of skin ulcer

**DOI:** 10.1080/07853890.2025.2525404

**Published:** 2025-07-05

**Authors:** Honglin Jia, Yang Tan, Hong Li, Xiaoqing Bu, Lingfei Li, Xia Lei

**Affiliations:** a Department of Dermatology, Army Medical Center (Daping Hospital), Army Medical University, Chongqing, PR China; b School of Public Health, Chongqing Medical University, Chongqing, PR China

**Keywords:** Skin ulcer, nomogram, predictive model, retrospective study

## Abstract

**Background:**

Wound healing is a complex process, and numerous factors affect the healing of skin ulcers.

**Objectives:**

In order to identify the factors associated with wound healing, it is necessary to establish a visualized predictive model for evaluating the risk factors of patients with skin ulcers and to validate its effectiveness.

**Methods:**

A retrospective observational study was conducted on 453 patients with skin ulcers admitted to the Dermatology ward of the Army Medical Center (Daping Hospital) in Chongqing, China, from January 2011 to July 2022. The nomogram was formulated according to a multivariate logistic regression analysis identifying seven potential predictors of prognosis, including age, area, pre-admission course, etiology, diabetes, medical treatment, and self-medication. This nomogram model was validated by bootstrap internal validation (1000 replicated samplings).

**Results:**

Logistic regression analysis showed that age, skin ulcer area, pre-admission course, etiology, comorbidity of diabetes, medical treatment, and self-medication were independently related to skin ulcer prognosis. These indicators were utilized to develop nomogram models. The predictive ability for skin ulcer prognosis was 0.814 based on the area under the curve values. The calibration curve showed a close match between the actual and predicted probabilities. Decision-making analysis demonstrated the clinical application value of this nomogram.

**Conclusion:**

The prediction nomogram developed in this study exhibits good accuracy in predicting the risk factors of skin ulcers and provides an objective tool for clinical staff to assess and target the risk factors concerning the prognosis of skin ulcers.

## Introduction

Skin ulcers, defined as injuries to the skin and deeper tissues (including the dermis and subcutaneous tissue), result from various etiological factors such as trauma (e.g. tearing, cuts, contusions), pressure, venous insufficiency, arterial ischemia, diabetes-related neurovascular complications and so on [1,2]. These ulcers are a prevalent condition in both daily life and clinical practice, affecting individuals across diverse populations at some point during their lifespan. Due to the heterogeneity in their etiology and clinical complexity, skin ulcers exhibit significant variability in recovery time, treatment strategies, and prognosis [3]. In severe cases, skin ulcers can severely impair patients’ quality of life and may even become life-threatening [4–6]. However, many patients fail to recognize the severity of their condition, leading to delayed treatment and poor outcomes. Additionally, inappropriate self-medication can exacerbate the condition, further delaying recovery and negatively impacting patients’ quality of life. Therefore, there is a critical need to establish a scientifically robust and accurate method for predicting the prognosis of skin ulcers at the early diagnostic stage. Such a tool could provide clinicians with innovative strategies for evaluating and selecting effective treatment options, ultimately improving patient outcomes.

Nomograms are widely recognized as straightforward, yet precise predictive tools developed based on real-world clinical data [7]. They enable the quantification of individual risk factors and their influence on disease progression, thereby supporting clinical decision-making [8]. Several studies have developed nomogram models to evaluate independent risk factors associated with specific types of skin ulcers, such as medical adhesive-related skin injuries [9,10]. However, these models are often limited to particular ulcer subtypes and lack generalizability across the broader spectrum of skin ulcers. Consequently, there is a need for a comprehensive nomogram model that can evaluate risk factors influencing the prognosis of skin ulcers more broadly. In this study, we aimed to develop and validate a retrospective nomogram model to predict the prognosis of skin ulcers based on clinically relevant variables. By leveraging clinical expertise and logical reasoning, this model, formulated by multivariate logistic regression and validated by bootstrap internal validation, aims to provide a more rational framework for establishing treatment plans at the early stages of skin ulcers. We hypothesize that such a tool will help shorten healing time, improve treatment efficacy, and enhance patients’ quality of life.

## Materials and methods

### Study population

We designed a retrospective study to create a nomogram for predicting the skin ulcer outcome of patients admitted to the ward of the dermatology department in the Army Medical Center (Daping Hospital). This study was approved by the Ethics Committee of the Army Medical Center. This study enrolled patients with skin ulcers of any etiology was included, such as those caused by skin diseases, external trauma, venous insufficiency, arterial ischemia, diabetes, or other systemic conditions. All patients who meet the inclusion criteria are included in the study. Patients lacking complete clinical and laboratory data were excluded from this study. Data was collected from the electronic medical records (EMRs) of patients admitted to the dermatology department of Daping Hospital between 2011 and 2022. A team of trained researchers, including dermatologists and data specialists, extracted the data using a standardized protocol to ensure consistency and accuracy. This study follows the Transparent Reporting of a multivariable prediction model for Individual Prognosis or Diagnosis (TRIPOD) guidelines (Supplementary Table 1).

### Inclusion and exclusion criteria

Inclusion criteria:

- Patients admitted for the first time to the dermatology department with skin ulcers of any etiology.
- Patients with complete clinical and laboratory data, including ulcer characteristics (size, location, duration), underlying conditions, and treatment history.

Exclusion criteria:

- Patients with incomplete medical records or missing essential data.

### Candidate variables

According to the pathogenic factors of skin ulcers and the risk factors that may affect the healing of skin ulcers [11,12], the Statistical Table of Patients with Ulcers was prepared, and the data of patients were obtained from electronic medical records. The collected data included age, gender, skin ulcer area, pre-admission course, healing time after admission, pathogen, comorbidities, microbiological examination, medical treatment, and self-medication. Ulcer area measurements were obtained from medical records, where trained clinicians documented the measurements using a standardized transparent film method. Biological samples of skin ulcers were acquired on the first day of admission for microbiological identification.

### Outcomes

Based on the previous study [13,14], skin ulcers that remained unhealed for over 1 month were identified as chronic ulcers. Therefore, patients were divided into the good prognosis group (healing time ≤30 days) and the poor prognosis group (healing time >30 days), and 30 days was used as the cut-off point.

### Methods building

Based on the criteria of inclusion and exclusion, 497 patients were enrolled in the study. The area of skin ulcers was first recorded to calculate and analyze the outliers. By Tukey’s statistical test, 44 outliers were kicked off (ulcer area 〈first quartile –1.5 × interquartile range *or* ulcer area〉 thire quartile +1.5 interquartile range), and 453 samples were included in the present study. For statistical analysis and clinical application, continuous variables, pre-admission course, were classified into ≤60 days and >60 days according to the median of collected data. The independent risk factors were recorded, compared, and validated by univariate analysis. In addition, a backward stepwise regression method was used to perform multivariate logistic regression analysis to screen for meaningful variables. The odds ratios (ORs) are presented with 95% confidence intervals (CIs). The nomogram model was generated using the R software package in order to facilitate the visualization of the results of the binary logistic regression. A variable with a significant value of *p* > 0.05 was excluded from the regression model.

### Model verification

The nomogram model was validated by bootstrap internal validation (1000 replicated samplings). Besides, to further estimate the calibration and efficiency of this predicted nomogram, we employed a series of analytical tools, namely the receiver operating characteristic curve (ROC), calibration curve, decision curve analysis (DCA), and clinical impact curve, respectively. These were obtained from the bootstrapping method. For ROC, the AUC (area under the curve) demonstrated the discrimination of this prediction model. Furthermore, the predictive capacity of the model was deemed superior when the bias-corrected curve was in closer alignment with the ideal curve. To gain further insight into the sensitivity and specificity of the model, the decision curve and impact curve were plotted.

### Statistical analysis

A medical record database was established using Excel. The statistical analysis was conducted using SPSS software (version 26.0) and the R software (version 4.2.2). The data were expressed as a number of cases (frequency) and analyzed using the Chi-square test. The Kolmogorov–Smirnov test was used to confirm the normal distribution of measurement data. The non-normally distributed measurement data were ­presented as ‘median (P25, P75)’ and analyzed by non-parametric tests. Binary logistic regression analysis was used for multivariate analysis, and the risk factors were screened by the backward stepwise method. All statistical analyses were performed using a two-tailed test, and a *p*-value of less than 0.05 was considered statistically significant. R software was used to construct this nomogram. In order to assess the discrimination of the nomogram model, the ROC, calibration curve, and decision curve were employed.

## Results

### Population

**Figure 1.**
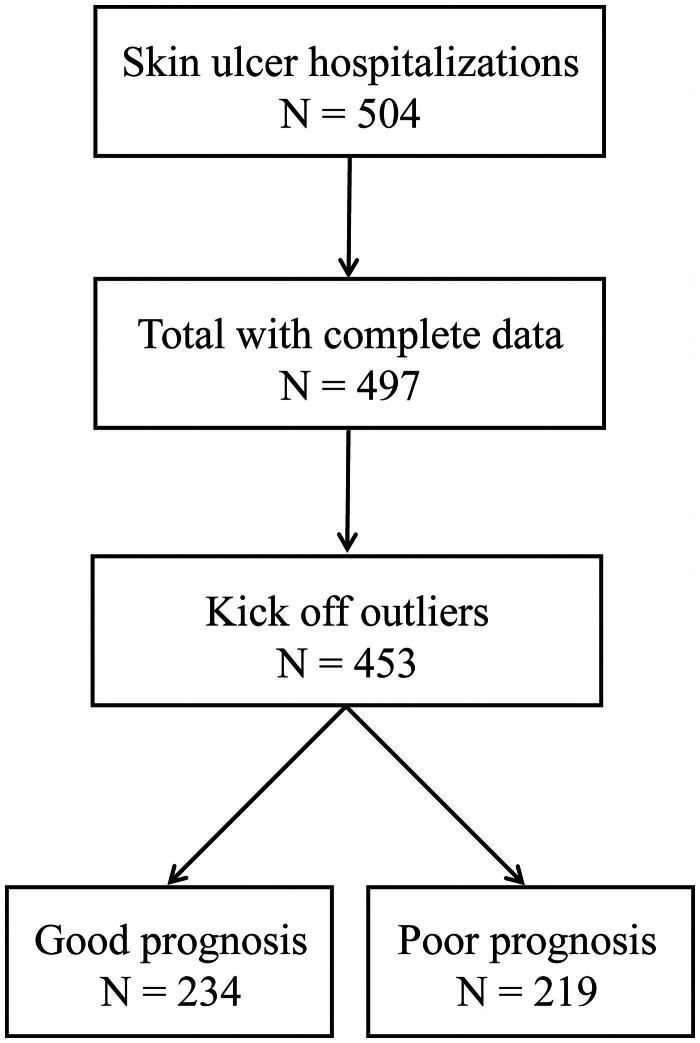
Schematic diagram of the study design.

We enrolled 497 patients admitted to the ward of the dermatology department in the Army Medical Center (Daping Hospital) between January 2014 and February 2022. Seven patients lacking complete clinical and laboratory data were excluded from this study. By Tukey’s statistical test, 44 outliers were kicked off, and 453 samples were proceeded in further statistical analysis and model construction, including 246 males and 207 females (Figure 1). Two hundred and thirty-four cases were considered a good prognosis, with a median age of 52 years, containing 134 males and 100 females. Besides, 219 patients, including 112 males and 107 females, were assigned to the poor prognosis group with a median age of 61.

### Comparison of disease characteristics

Demographic, clinical, and bacteriological characteristics of included patients were summarized in Table 1. To analyze the independent risk factors that affect the prognosis of skin ulcers, we used univariate analysis to evaluate the disease characteristics of skin ulcers from different aspects. Briefly, age, skin ulcer area, etiology, comorbidity, bacterial infection, pre-admission course, and self-medication were influencing factors of the skin ulcer prognosis (*p* < 0.05). However, there was no statistical significance for the gender, medical treatment and skin ulcer position by univariate analysis. Patients with poor prognosis demonstrated significantly higher age, larger skin ulcer areas, longer pre-admission course, and more complex bacterial infections. Meanwhile, the pre-medical experience of patients also plays a vital role in skin ulcer prognosis. Patients’ more pre-hospital medical experience indicated more emphasis on the diseases so that they would go to the hospital on time. However, there is no statistical significance in medical treatment (*p* = 0.051). We think that the small size of our population is the main reason. In the etiology, trauma is the most common reason for skin ulcers, wherever in good or poor prognosis group, it was pertaining to skin ulcer prognosis with a *p* < 0.001. Other etiology includes pyoderma gangrenosum (PG), panniculitis, skin carcinoma and so on. PG is a rare neutrophilic dermatosis that shows rapidly developing and painful skin ulcers hallmarked by undermined borders and peripheral erythema [15]. As evidenced by Table 1, PG is an independent risk factor for influencing prognosis. Even though there is no significant difference in skin cancer, it may be attributed to the current limited sample size. Nontuberculous mycobacteria (NTM) species, classified by their slow or rapid growth rates, can cause various illnesses, from skin ulceration to severe pulmonary and disseminated infective disease [16]. Among the bacteria species, the NTM is a risk factor with a poor prognosis with statistical significance.

**Table 1.** Baseline characteristics of patients with skin ulcers.

Variables	Good prognosis *n* = 234	Poor prognosis *n* = 219	Statistical value	*P*
Age (median [IQR])	52 (37–64)	61 (49–72)	−5.137	<0.001
Male (%)	134 (57.3)	112 (51.1)	1.709	0.191
Area (median [IQR])	3.7 (2.0–6.4)	8.0 (4.0–14.0)	−7.668	<0.001
Pre-admission course (%)			28.862	<0.001
≤60 days	168 (71.8)	103 (47.0)		
>60 days	66 (28.2)	116 (53.0)		
Position (%)			9.093	0.054
Lower limbs	174 (74.4)	158 (72.1)		
Upper limbs	12 (5.1)	20 (9.1)		
Face	9 (3.8)	3 (1.4)		
Torso	38 (16.2)	32 (14.6)		
Two or more parts	1 (0.4)	6 (2.7)		
Etiology (%)			12.764	<0.001
Trauma	218 (93.2)	180 (82.2)		
Other^a^	16 (6.8)	39 (17.8)		
Comorbidity (%)				
Diabetes	16 (6.8)	29 (13.2)	5.186	0.023
Vascular disease	20 (8.5)	44 (20.1)	12.427	<0.001
Carcinoma	0	8 (3.7)	6.723	0.010
Bacterial infection (%)			9.332	0.009
Yes	103 (44.0)	124 (56.6)		
No	60 (25.6)	53 (24.2)		
Uncultured	71 (30.3)	42 (19.2)		
Bacterial species (%)			7.626	0.022
None or uncultured	131 (56.0)	95 (43.4)		
One	93 (39.7)	115 (52.5)		
Two	10 (4.3)	9 (4.1)		
Bacterial category (%)				
S. aureus	23 (9.8)	19 (8.7)	0.179	0.672
P. aeruginosa	26 (11.1)	20 (9.1)	0.485	0.486
MRSA	0	3 (1.4)	1.480	0.224
NTM	0	41 (18.7)	28.525	<0.001
Other	64 (27.4)	50 (22.8)	1.227	0.268
Medical treatment (%)			3.812	0.051
Yes	141 (60.3)	112 (52.1)		
No	93 (39.7)	107 (48.9)		
Self-medication (%)			5.440	0.020
Yes	80 (34.2)	53 (24.2)		
No	154 (65.8)	166 (75.8)		

Count data were expressed as ‘number of cases (frequency),’ and the measurement data was presented as ‘median (P25, P75)’. S. aureus, Staphylococcus aureus; P. aeruginosa, Pseudomonas aeruginosa; MRSA, methicillin-resistant Staphylococcus aureus; NTM, non-tuberculous mycobacteriosis.

^a^Other etiology including pyoderma gangrenosum (PG), panniculitis, skin carcinoma and so on.

### Multivariate analysis of skin ulcer risk factors and construction of nomogram model

To construct the nomogram model, we screened the most informative variables from the risk factors with binary logistic regression analyses by the backward stepwise regression method [17]. The result demonstrated that age (OR, 1.039; 95% CI, 1.025 to 1.054; *p* < 0.001), skin ulcer area (OR, 1.146; 95% CI, 1.101 to 1.193; *p* < 0.001), pre-admission course (OR, 3.078; 95% CI, 1.928 to 4.915; *p* < 0.001), etiology (OR, 3.341; 95% CI, 1.621 to 6.887; *p* = 0.001), and comorbidity of diabetes (OR, 2.253; 95% CI, 1.071 to 4.742; *p* = 0.032) were independently related to a higher risk of poor prognosis, whereas medical treatment (OR, 0.351; 95% CI: 0.227 to 0.576; *p* < 0.001) and self-medication (OR, 0.266; 95% CI: 0.155 to 0.457; *p* < 0.001) were independently related to decreased odds of poor prognosis (Table 2).

**Table 2.** Multivariate logistic regression models to analyze the predictive factors concerning skin ulcers.

Variables	B	OR	95% CI	*P*
Age	0.039	1.039	1.025–1.054	<0.001
Area	0.136	1.146	1.101–1.193	<0.001
Pre-admission course	1.124	3.078	1.928–4.915	<0.001
Etiology	1.206	3.341	1.621–6.887	0.001
Diabate	0.812	2.253	1.071–4.742	0.032
Medical treatment	−1.030	0.351	0.227–0.576	<0.001
Self-medication	−1.323	0.266	0.155–0.457	<0.001

B: Regression coefficient; OR: odds ratio; CI: confidence interval.

**Figure 2.**
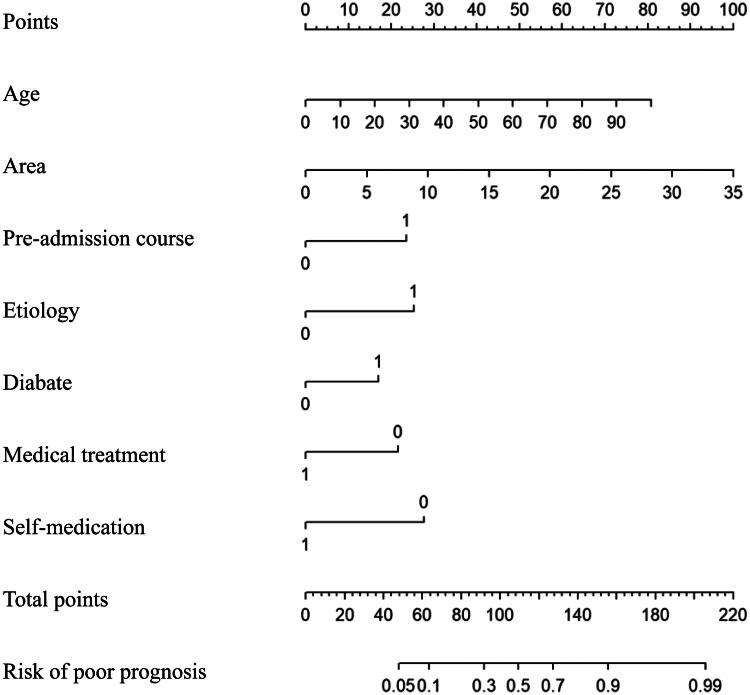
Prediction nomogram model for the prognosis of patients with skin ulcers.

We utilized these independent influencing factors (*p* < 0.05) in logistic regression to develop a nomogram model [18]. As shown in Figure 2, the point values for each variable have been added together to produce the total point value. A higher total score suggests a higher probability of achieving a poor prognosis during the recovery of a skin ulcer. The highest score in the nomogram model was 35 cm^2^ of skin ulcer area. Age was the second highest score in our nomogram model; a higher age of skin ulcer patients indicated a higher possibility for a signature of poor prognosis. For the etiology, trauma, the most common reason causing skin ulcers, signified a better prognosis compared with other etiology. Notably, comorbidity of diabetes was related to the poor prognosis of skin ulcers in our nomogram model. Pre-hospital experience was also a critical variety in our nomogram model since it could indicate the severity of patients’ skin ulcers. In our nomogram model, both the medical treatment and self-medication were associated with a lower risk of poor prognosis of skin ulcers.

### Evaluation of the accuracy and predictive effectiveness of the nomogram model

**Figure 3.**
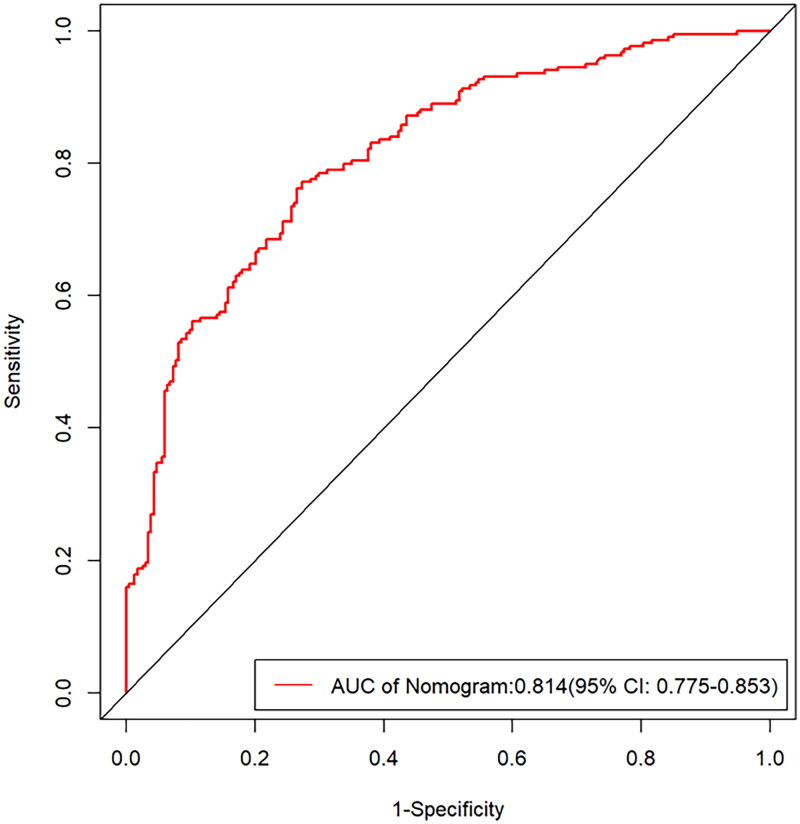
Receiver operating characteristic curves for the nomogram model. AUC, the area under the curve.

**Figure 4.**
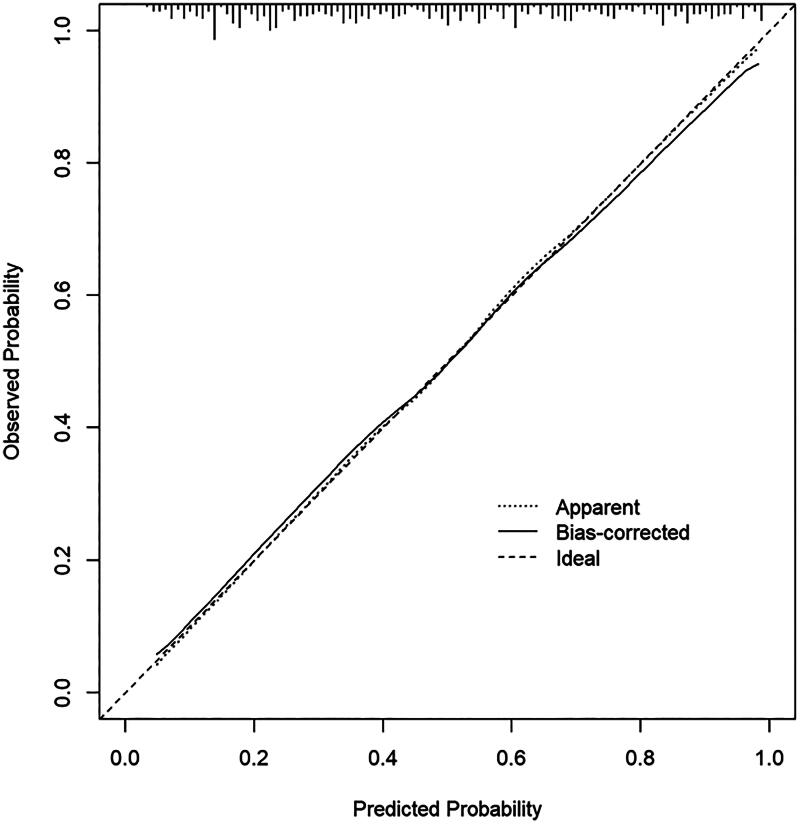
Calibration curves for the nomogram model.

**Figure 5.**
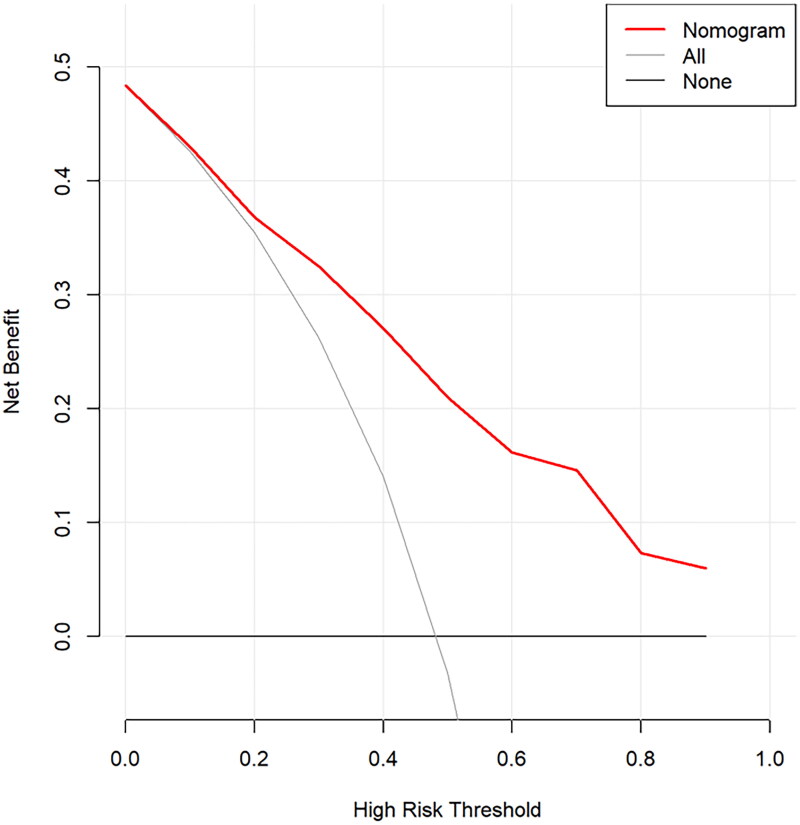
Decision curve analysis demonstrating the net benefit associated with the use of this nomogram model.

The ROC curve evaluated the discrimination capacity of the nomogram model [19]. The C index of the prediction model was 0.814, with a 95% confidence interval of 0.775–0.853, indicating sufficient discrimination ability and high accuracy (Figure 3). Besides, the calibration curve was constructed using the bootstrap (1000 bootstrap resamples) method. As depicted in Figure 4, the bias-corrected calibration curve was close to the idea curve, suggesting satisfactory concordance between the observation and predictions. To assess the clinical application potential of the predictive model [20], the decision curve analysis (DCA) was developed (Figure 5). The clinical decision curve showed the clinical benefits provided by the nomogram model at the decision thresholds between 0 and 0.9 (Figure 5).

## Discussion

Due to the complexity and diversity of skin ulcers, predicting prognosis and providing timely and effective medical care based on the patient’s condition is challenging. Nomogram model is a widely used predicting model helping clinicals to evaluate diseases. However, there is still lack of a nomogram model using for skin ulcers. In the present study, we firstly developed a predictive nomogram model to detect the risk factors of skin ulcers and give medical staff an objective tool to provide timely and appropriate medical intervention for patients.

In our study, we finally collected 453 medical records of skin ulcer patients through a retrospective analysis. Based on the characteristics of skin ulcers, the data we collected included age, gender, skin ulcer area, pre-admission course, healing time after admission, etiology, comorbidities, bacteriological examination, medical treatment, and self-medication. By univariate analysis and further multivariate analysis, we finally enrolled seven potent independent risk factors that influence the prognosis of skin ulcers to construct a nomogram model (age, skin ulcer area, pre-admission course, etiology, comorbidity of diabetes, medical treatment, and self-medication). The ROC curve, C index, and calibration curve indicated the excellent discrimination and predictive ability of our nomogram. Besides, the clinical decision curve and impact curve demonstrated that our model accurately predicted the impact factors of skin ulcer prognosis, which could guide the clinical practices to intervene in skin ulcers properly.

The highest score in the model was skin ulcer area, indicating that patients with larger trauma areas were related to a higher risk of poor prognosis. The skin ulcer with large area could not only impede the recovery process but also renders the wound more vulnerable to additional risk factors, such as bacterial infections, reduced mobility, and limited therapeutic interventions, which led to a poor prognosis of skin ulcers. Age emerged as another pivotal prognostic factor for skin ulcer outcomes in our nomogram model. The age-related alterations in dermal and epidermal, such as degradation of dermal collagen fibrils, impaired physiological function of skin resulting in the poor prognosis of skin ulcers [21]. In addition, the diminished immune response of elderly patients made the skin ulcer more susceptible to bacterial infection, which further deteriorated the skin ulcers [22].

Additionally, our investigation identified etiology as a significant determinant that impacting skin ulcer prognosis. In the present study, PG, panniculitis, and skin carcinoma were considered and analyzed. Notably, only PG was correlated with poor skin ulcer prognosis. Currently, many researchers have hypothesized that immune dysfunction contributes to PG [23]; the overactive immune reaction results in excessive secretion of inflammatory mediators, which impede skin ulcer recovery and lead to a poor prognosis [24]. Thus, timely interventions to ameliorate the hyperactive immune response in PG-induced skin ulcers are necessary. However, there was no statistically significant difference in either panniculitis or skin carcinoma in affecting skin ulcers’ prognosis, which is contrary to common sense. The reason may be attributed to the limited sample size.

Diabetes was also another crucial factor contributing to poor skin ulcer prognosis in the present data. One of the possible reasons is the reduced nervous responses [25]. It’s hard for diabetes patients to feel the pain and realize the formation of skin ulcers, further making it unable to be treated promptly [26]. Besides, the weak immune system of diabetes patients was also a critical reason for making skin ulcers hard to heal [27]. Additionally, dysregulated blood glucose levels in diabetic patients often indicate peripheral neurovascular abnormalities, resulting in compromised blood and oxygen supply to the skin ulcer site [28]. The diminished perfusion induces insufficient nutritional and oxygen levels in the skin ulcer site to support regenerative healing, leading to a poor prognosis [29]. Therefore, managing blood glucose levels is crucial for healing the skin ulcer in diabetic patients.

Prehospital experience also greatly influenced skin ulcer prognosis. A protracted course often signifies inherent challenges in skin ulcer healing, while the patients’ inattention and irregular disposition further exacerbate the skin ulcer progression. The medical experience not only provided timely medical intervention that promoted the skin ulcer’s recovery but also indicated that patients had paid enough attention to their diseases. Similarly, self-medication signified the importance of patients’ attachment to skin ulcers. The self-medication also partly retarded the progress of skin ulcers. But we do not suggest that every skin ulcer patient to have medication on themselves. Informal medical disposal, such as using traditional Chinese herbal grindings and abuse of antibacterial, can also impede the healing of skin ulcers or even induce the infection of drug-fast bacteria, which will deteriorate the skin ulcers. Thus, enhancing patients’ knowledge of skin ulcers is an imperative manner to reduce the unfavorable prognosis of skin ulcers.

Several study limitations should be noted. The retrospective design was constrained by incomplete medical records, and our model did not address etiology-specific variations (e.g. diabetic vs venous ulcers) that stratified models might better capture. Additionally, the single-center design and limited sample size may affect generalizability. Future multicenter studies with etiology-stratified larger cohorts are needed to validate these findings.

In summary, we identified seven factors significantly associated with poor skin ulcer prognosis by constructing the nomogram model. Our predictive model will help clinicians promptly identify individuals with increased poor skin ulcer prognosis, promoting timely and targeted interventions based on their unique skin ulcer characteristics to improve the prognosis and quality of patients’ lives.

## Conclusion

This study identified age, area, pre-admission course, etiology, comorbidity, bacterial infection, medical treatment, and self-medication as risk factors of poor prognosis in skin ulcer patients. The risk nomogram prediction model we developed will be served as a valuable tool for clinical practice in identifying high-risk skin ulcer patients with high efficiency in order to provide personalized medical care.

## Supplementary Material

Supplementary Table 1 Tripod.docx

## Data Availability

Data are available on request from the corresponding authors.
